# Cystoid Macular Lesions in Inherited Retinal Diseases: Prevalence, Characteristics, and Genetic Associations in a Hungarian Cohort

**DOI:** 10.3390/genes16101212

**Published:** 2025-10-14

**Authors:** Barbara Asboth, Alessandra Sanrocco, Barbara Besztercei, Balazs Lesch, Agnes Takacs, Rita Vamos, Balazs Varsanyi, Andras Vegh, Krisztina Knezy, Viktoria Szabo, Zoltan Zsolt Nagy, Ditta Zobor

**Affiliations:** Department of Ophthalmology, Semmelweis University, Budapest 1085, Hungary; asboth.barbara@semmelweis.hu (B.A.);

**Keywords:** inherited retinal diseases (IRD), cystoid macular lesion (CML), cystoid macular edema (CME), optical coherence tomography (OCT), gene therapy, retinitis pigmentosa

## Abstract

**Background/Objectives**: Cystoid macular lesion (CML) is a treatable cause of central vision loss in inherited retinal diseases (IRDs). We aimed to determine the frequency of CML in a large Hungarian IRD cohort and examine associations with causative genes. **Methods**: This longitudinal, retrospective, monocentric study included patients with genetically confirmed IRD identified from our database. Targeted next-generation sequencing (351-gene panel) and comprehensive ophthalmic evaluation were performed, including best-corrected visual acuity (BCVA) and spectral domain optical coherence tomography (SD-OCT). CML was defined as intraretinal hyporeflective spaces with well-defined borders visible on at least two B-scans within the SD-OCT macular volume and was categorized as cystoid macular edema (CME) or non-CME. **Results**: We enrolled 430 patients with genetically confirmed IRDs. CML was detected in 93 eyes of 57 patients. Mean age at OCT was 36.6 ± 18.7 years (range, 3–76); 32 were male (56.1%). Inheritance patterns were autosomal recessive in 24 (42.1%), X-linked in 19 (33.3%), and autosomal dominant in 14 (24.6%). Frequently implicated genes were *RS1* (12/57), *USH2A* (7/57), *NR2E3* (7/57), *PRPF31* (4/57), *RPGR* (4/57), and *RHO* (4/57). CME predominated in retinitis pigmentosa (32/57, 56%), with mean BCVA 0.44 ± 0.29 (decimal) and central retinal thickness (CRT) 401 ± 181 µm. Non-CME CML occurred in 25/57 (44%)—notably in X-linked retinoschisis and enhanced S-cone syndrome—with BCVA 0.40 ± 0.23 and CRT 465 ± 258 µm. BCVA did not correlate with CRT (r_S_ = 0.18). **Conclusions**: CML occurred in 13.2% of patients within a large Hungarian cohort of genetically confirmed IRDs. Patients with IRD—mainly RP—are at higher risk for CML. Gene therapy is promising for retinal diseases, but CMLs can compromise effectiveness. Reducing and managing CME before gene therapy corroborates retinal stability and the functional state essential for the proper delivery and penetration of corrective genes to the target cells.

## 1. Introduction

Inherited retinal diseases (IRDs) are a diverse group of congenital degenerative conditions affecting the retina and/or retinal pigment epithelium (RPE). Affecting over two million individuals worldwide, these diseases arise from genetic and environmental factors that disrupt retinal development and function. IRDs are classified based on the primary site of manifestation (cones, rods, and RPE), the presence of extraocular symptoms, age of onset (congenital or late-onset), disease progression (stationary or progressive), mode of inheritance, and the underlying genetic defect [1].

The genetic landscape of IRDs is complex, with over 300 identified causative genes, and this number is continually expanding. A single gene mutation can result in various IRDs, while a single IRD can be caused by mutations in multiple genes, highlighting both clinical and genetic heterogeneity. This complexity poses significant challenges for genetic testing. However, genotype clarification is crucial for accurate diagnosis, genetic counseling, and the development of gene-specific therapies [2,3,4].

A prevalent manifestation in IRDs is macular abnormality, specifically cystoid changes, referred to as cystoid macular lesions (CMLs). Several IRDs, including retinitis pigmentosa (RP), juvenile X-linked retinoschisis (XLRS), enhanced S-cone syndrome (ESCS), choroideremia, and gyrate atrophy, have been associated with the presence of CML. CMLs can significantly affect central visual acuity, particularly in patients who may already have severely constricted peripheral visual fields. Given the clinical and therapeutic relevance of CML—particularly in the context of emerging gene therapies—accurate detection and understanding of its genetic associations is essential [5].

**Figure 1:**
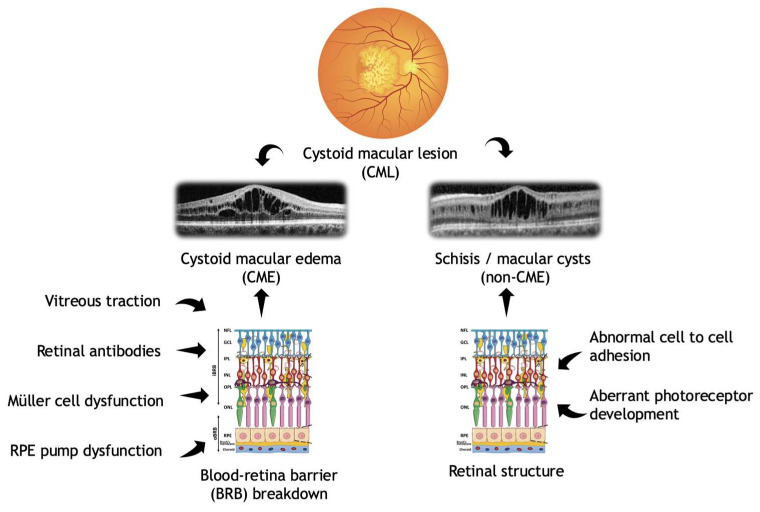
Pathophysiology of cystoid macular lesions subclassified into CME and non-CME lesions (created using BioRender.com).

While it is well established that CMLs can contribute to visual impairment, the underlying pathophysiological mechanisms remain an active area of research (Figure 1).

Several hypotheses have been proposed, including breakdown of the blood–retinal barrier (BRB), dysfunction of the retinal pigment epithelium (RPE) pump, and Müller cell edema and dysfunction. Retinal autoantibodies and vitreomacular traction have also been implicated as potential contributing factors, although further research is needed to clarify their roles [6].

CML can be further subclassified into cystoid macular edema (CME) and non-CME CML, often referred to as macular cysts or schisis. While both forms involve fluid accumulation within the retina, they differ in pathophysiology, anatomical localization, and clinical presentation. CME is typically associated with breakdown of BRB, resulting in intraretinal fluid accumulation predominantly in the inner nuclear layer, often presenting ophthalmoscopically with characteristic fovea light and macular reflex alteration. Non-CME macular cysts may develop due to disruption of retinal architecture in the macular region, with fluid localized to the outer plexiform layer (Henle’s layer), often appearing ophthalmoscopically as radially oriented ovoid spaces [7].

Optical coherence tomography (OCT) provides objective, high-resolution cross-sectional imaging of the retina, making it an invaluable tool for the diagnosis, characterization, and monitoring of CMLs. The advent of OCT enabled non-invasive assessment of CMLs, reducing the need for routine fluorescein angiography (FA) [8].

We present a retrospective analysis of a large Hungarian cohort of patients with genetically confirmed IRDs. The primary objective of this study was to determine the prevalence of CMLs, as assessed by OCT, in this well-characterized IRD cohort and to identify potential associations between the presence and characteristics of CMLs and specific causative genes. The elucidation of such genotype–phenotype correlations may provide valuable insights into the pathogenic mechanisms underlying CML formation in IRDs and potentially inform the development of targeted therapeutic strategies.

## 2. Materials and Methods

This retrospective study was conducted on data retrieved from the electronic medical records of patients with IRDs who were evaluated at the Department of Ophthalmology, Semmelweis University between 2021 and 2024. The study protocol was approved by the Review Board of the Medical Research Council, Health Ministry of Hungary (No. 28002-6/2021) and adhered to the tenets of the Declaration of Helsinki. A signed informed consent was obtained from all patients with a suspected diagnosis of IRD. The database comprised comprehensive clinical records, multimodal imaging data, and genetic test results of patients with suspected IRDs.

### 2.1. Ophthalmic Examination

We included eyes with documented CMLs on OCT from patients with a confirmed genetic diagnosis of IRD. Patients lacking OCT imaging were excluded from the analysis. Spectral domain OCT imaging was performed using either the Heidelberg Spectralis (Heidelberg Engineering, Heidelberg, Germany) or RTVue (Optovue Inc., Fremont, CA, USA) systems. CML was defined as the presence of intraretinal hyporeflective spaces with distinct borders visualized on at least two B-scan lines on OCT. CMLs were subcategorized as either cystoid macular edema (CME) or non-CME CML based on disease etiology. Following manual adjustment for foveal centration and correction of retinal layer segmentation errors, central retinal thickness (CRT) was measured in µm. CFT was defined as the vertical distance, in micrometers (µm), between the internal limiting membrane (ILM) and the outer boundary of the retinal pigment epithelium (RPE) at the fovea.

All patients underwent a standardized comprehensive ophthalmic examination, including assessment of best-corrected visual acuity (BCVA) using a Snellen chart, slit-lamp biomicroscopy, dilated fundus examination, and OCT imaging.

### 2.2. Genetic Testing

Targeted next-generation sequencing (NGS) was performed using custom panels encompassing 351 known IRD-associated genes. Genetic testing results were categorized according to standard classifications, including variants of uncertain significance (VUS), likely benign, benign, likely pathogenic, and pathogenic. For the purpose of this study, only variants classified as likely pathogenic or pathogenic were considered confirmatory for the genetic diagnosis of IRD [9]. In occurrences where a genetic variant is classified as a VUS, the confirmation of the final diagnosis was supported by considering family history, family variant testing, clinical phenotype, and additional genetic evidence, such as the absence or very low frequency in extensive population databases of healthy individuals, as well as functional studies demonstrating a detrimental impact on gene or protein function.

### 2.3. Statistical Analysis

The statistical analysis of the data was conducted by using the JMP 18 statistical software (SAS Institute, Cary, NC, USA). Data normality was assessed by evaluating histogram plots. Correlation parameters were calculated using Spearman correlations due to non-parametric distribution.

## 3. Results

### 3.1. IRD Cohort: General Findings

Out of the 519 instances of IRD, genetic confirmation of the diagnosis was achieved in 430 patients. Consequently, 89 patients with negative genetic results but suspected IRD were excluded from further analysis in this study. In the remaining 430 cases, CML was detected in 93 eyes of 57 patients, involving 19 distinct genes out of a total of 80 genes identified across the entire cohort. Table 1 and Table 2 summarize the clinical characteristics.

**Figure 2:**
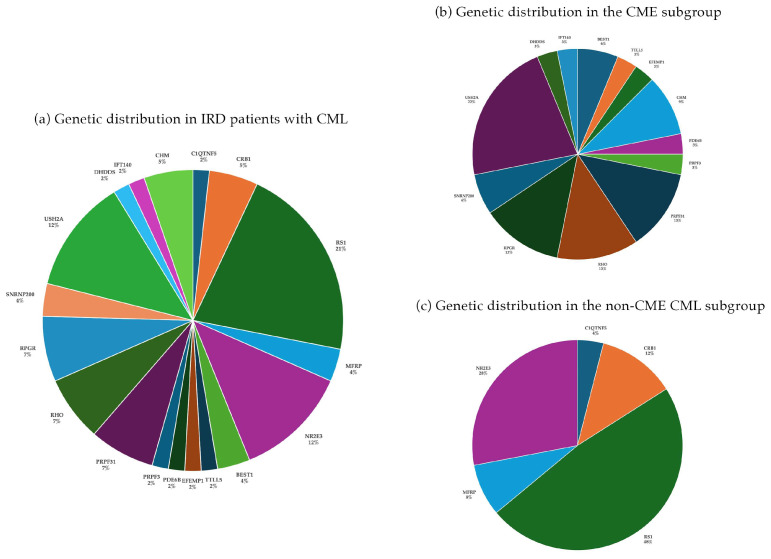
Genetic distribution in the overall IRD cohort of the study (**a**) and in the two subgroups: (**b**) patients with cystoid macular edema (CME) and (**c**) patients with non-CME (schisis-like) cystoid macular lesions (CML).

Among the 57 patients with CML, 21 (36.8%) had unilateral involvement, while 36 (63.2%) presented with bilateral CML. The mean age at the time of OCT evaluation was 36.6 ± 18.7 years (range: 3–76 years), and 32 patients were male (56.1%). The average BCVA across all patients was 0.42 ± 0.26 (decimal), and the mean CRT was 430 ± 220 µm. In general, an autosomal dominant trait was identified in 14 patients (24.6%), autosomal recessive in 24 patients (42.1%), and X-linked inheritance in 19 patients (33.3%). In the AD group, 9 of 14 patients (64.3%) showed bilateral CML, while 5 patients (35.7%) had unilateral involvement. Among the AR group, 13 of 24 patients (54.2%) exhibited bilateral CML, and 11 (45.8%) were unilaterally affected. In the X-linked group, bilateral CML was present in 14 of 19 patients (73.7%), while 5 patients (26.3%) had unilateral CML. The most common genes included *RS1* (12/57), *USH2A* (7/57), *NR2E3* (7/57), *PRPF31* (4/57), *RPGR* (4/57), and *RHO* (4/57). These six genes accounted for 66% (38/57) of all cases. (Figure 2). Certain genes, which were more frequently represented within the cohort, including *ABCA4* (97/430), *RPE65* (14/430), *PRPH2* (11/430), or *EYS* (8/430), were not associated with CML in any cases. A discrepancy was observed between CRT and BCVA, indicating that visual acuity was not reliably predictable based on the neurosensory retinal thickness at the fovea in these patients (Spearman correlation coefficient r_S_ = 0.18).

### 3.2. CME Cohort

We further investigated the two subcategories based on the underlying pathomechanisms and selected the following occurring genotypes into this group: *BEST1*, *CHM, DHDDS*, *EFEMP1*, *IFT140*, *PDE6B*, *PRPF3*, *PRPF31*, *RHO*, *RPGR*, *SNRNP200*, and *USH2A*. CME was the predominant presentation among patients with CML, occurring in 32 out of 57 cases (56%), and was primarily associated with RP. Within our cohort, we identified 25 patients diagnosed with RP accompanied by concomitant CME, spanning various genetic subtypes and inheritance patterns, affecting a total of 42 eyes. This group comprised 15 females and 10 males. In the remaining subset of seven patients within the CME group, additional genes, such as *BEST1*, *CHM, TTLL5*, and *EFEMP1*, were identified (Figure 2b). These genes appeared in singular or a few cases, thereby illustrating a broad phenotypic spectrum that includes conditions such as Best disease, choroideremia, cone–rod dystrophy, and autosomal dominant drusen.

Patients with CME exhibited a mean BCVA of 0.44 ± 0.29 (decimal), and their mean CRT measured 39 ± 178 µm. The mean age of the patients was 41 years, with a range of 19 to 67 years. CME was bilateral in 17 patients (68%) and unilateral in 8 patients (32%). Among patients with autosomal recessive (AR) RP (*n* = 10), CME was bilateral in seven and unilateral in three cases. In the autosomal dominant (AD) group (*n* = 11), eight patients presented with bilateral CME and three patients with unilateral involvement. Among the four patients with X-linked RP (XLRP, three males and one female) due to disease causing variants in the *RPGR* gene, CME was bilateral in two male patients and unilateral in the other two patients. Among the AR group, the most frequently implicated gene was *USH2A*, found in seven patients, followed by single cases associated with *DHDDS*, *IFT140,* and *PDE6B*. In the AD cohort, disease-causing variants were identified in *RHO* (*n* = 4), *PRPF31* (*n* = 4), *SNRNP200* (*n* = 2), and *PRPF3* (*n* = 1). Within this subgroup, the correlation between BCVA and CRT was observed to be weak, with an r_S_ = 0.34 (Table 3).

**Figure 3:**
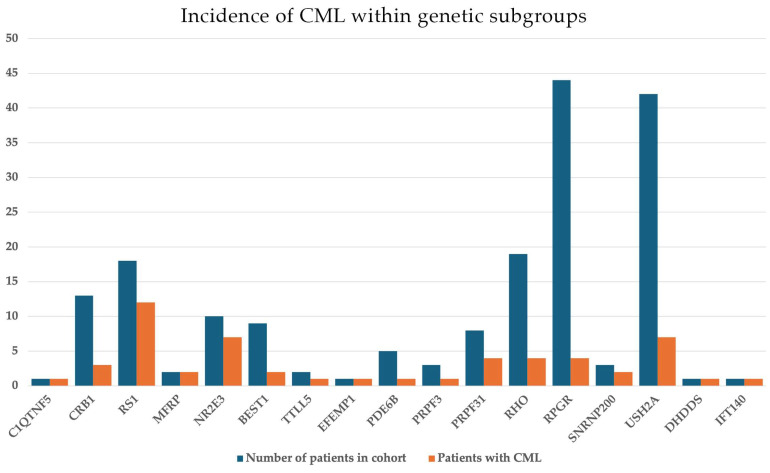
Incidences of cystoid macular lesions (CML) within the different genetic subgroups observed in our study. The blue columns show the total number of patients in each genotype group, while orange columns demonstrate the number of patients affected with CML within each group.

The mean BCVA in RP-CME patients was 0.42 ± 0.24 in the right eye (OD) and 0.43 ± 0.3 in the left eye (OS). CRT was measured 419 ± 160 µm and 438 ± 203 µm in OD and OS, respectively. CRT values were highest among *RHO*-associated RP cases, with some patients presenting with macular thickness exceeding 800 µm, suggesting significant intraretinal fluid accumulation. As in the CME subgroup, a similarly weak correlation was found between BCVA and CRT with an r_S_ = 0.36. Notably, the incidence of CME was more prevalent in RP cases exhibiting an AD inheritance pattern overall (11/32, 34%) and was also significant when assessed for each genotype. Specifically, CME manifested in 21% of all *RHO* cases (4/19), 50% of *PRPF31* cases (4/8), and 66% of *SNRNP200* cases (2/3). Conversely, within the AR-RP and X-linked RP (XLRP) cohorts, CME was less common, occurring in 20% of AR-RP cases (10/49) and 14% of XLRP cases (7/50), respectively. Specifically, AR-RP *USH2A* cases showed an incidence of 16.6% (7/42) and XLRP linked to *RPGR* mutations showed an incidence of 9% (4/44) (Figure 3). While *ABCA4* (*n* = 97), *RPE65* (*n* = 14), and *PRPH2* (*n* = 11) accounted for a substantial part of the entire cohort, no instances were recorded in IRD cases linked to these genes. An estimation of 12.4% of CME incidence in all RP cases was calculated (25/201).

### 3.3. Non-CME CML (Schisis-like) Cohort

In contrast, the remaining 25 patients (44%) presented with non-CME CML (Table 4).

**Figure 4:**
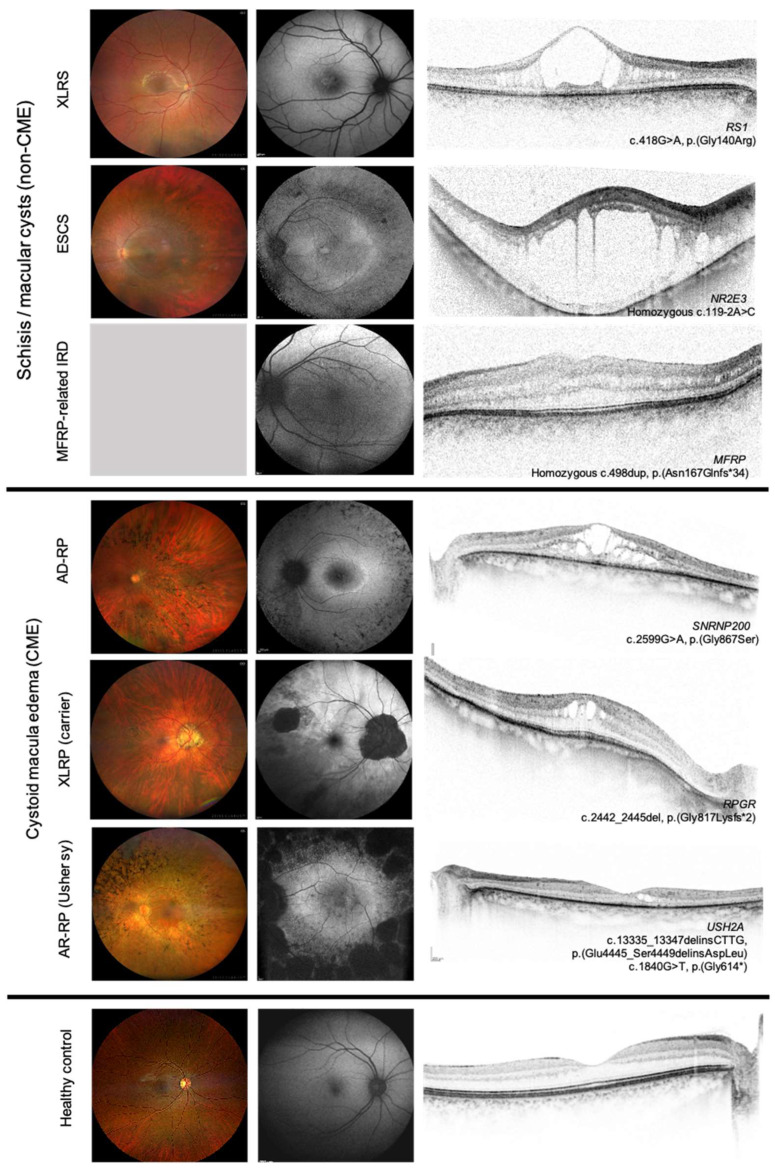
Retinal imaging in inherited retinal diseases (IRDs) showing different forms of cystoid macular lesions (CMLs). From left to right: color fundus images (Clarus 700, Carl Zeiss AG, Oberkochen, Germany), fundus autofluorescence and OCT images (Spectralis, Heidelberg Engineering, Heidelberg, Germany). The IRD due to mutations in *MFRP* includes the following: nanophthalmus, retinal degeneration, foveoschisis, and optic disc drusen. AD-RP: autosomal dominant retinitis pigmentosa, AR-RP: autosomal recessive retinitis pigmentosa, ESCS: enhanced S-cone syndrome, XLRP: X-linked retinitis pigmentosa, and XLRS: X-linked retinoschisis. The lower panel shows images of a healthy control for comparison.

These cases were linked to other forms of IRDs, most notably X-linked retinoschisis (XLRS) and enhanced S-cone syndrome (ESCS). The two causative genes together, *RS1* and NR3E3 were responsible for 33% (19/57) of all cases in our patient cohort. In this group (Figure 2c), BCVA was 0.4 ± 0.23 and CRT was 465 ± 258 µm. CML was bilateral in 17 patients (68%) and unilateral in eight patients. The mean age at the time of OCT evaluation was 33 ± 23 years (range: 4–76 years), and 18 patients were male. A limited number of cases involved additional genes, including *CRB1* (3/57), *MFRP* (2/57), and *C1QTNF5* (1/57). The majority of cases presenting with CML were associated with *RS1* in the overall cohort, with the mean BCVA being 0.36 ± 0.18 and 0.40 ± 0.17, and correspondingly, CRT being 416 ± 133 µm and 524 ± 168 µm in the right and left eye, respectively. The mean age at the time of OCT evaluation was 22 ± 18.8 years. In the subgroup of patients with non-CME CML, no statistically significant correlation was found between BCVA and CRT (r_S_ = −0.05). Similarly, patients diagnosed with XLRS demonstrated an absence of a meaningful correlation between these two parameters (r_S_ = −0.16). Figure 4 summarizes the morphological findings of IRD patients with CML.

In addition to the subgroup analysis based on the underlying pathomechanism, we also evaluated BCVA and CRT values for each mode of inheritance (AD, AR, or X) and found no statistically significant differences between these subgroups (BCVA was 0.41 ± 0.3, 0.44 ± 0.3 and 0.41 ± 0.2 decimal; CRT was 391 ± 210, 440 ± 276 and 445 ± 150 µm in each inheritance mode, respectively).

## 4. Discussion

The pathogenesis of CML in IRDs is not fully understood. Contributing factors include BRB breakdown leading to retinal fluid accumulation (CME), RPE dysfunction affecting fluid transport, and Müller cell dysfunction disrupting retinal homeostasis. Retinal architectural disruptions due to cell adhesion defects can further promote fluid build-up and cyst formation. These complex interactions underscore the intricacy of CML development in IRDs.

Depending on the underlying pathophysiology, CML can be further subcategorized into CMEs and non-CME CMLs (macular cysts or schisis). Macular cysts observed in retinal dystrophies may arise due to the disruption of the retinal architecture within this region. The macular region has a predilection to develop these changes because of the absence of multiple supportive retinal layers. Retinoschisin, encoded by the *RS1* gene is expressed in all retinal neurons except horizontal cells during development, and later in mature retina by photoreceptors and bipolar cells. It plays a key role in maintaining retinal structure and ion balance by binding to Na+/K+ -ATPase pumps [10,11]. Disruption of this interaction may lead to extracellular fluid buildup in schitic spaces in XLRS, but the exact mechanism of cystoid space development and macular thickening is uncertain [12]. In patients affected by ESCS, the fundus can have variable manifestation on imaging, but the most frequent are white-yellow dots and nummular pigmentation along the vascular arcades, both associated with a limited disease progression. Several studies have shown schisis-like foveal changes with no visible leakage on, suggesting that the cystoid spaces are not a consequence of inner or outer BRB damage [13]. The etiology of cystic cavities in ESCS may be attributed to synaptic modifications associated with laminar structural disorganization of the retina, which occurs as a response to an excess of S-cone photoreceptors, all of which are consequences of pathogenic *NR2E3* gene variants [13,14,15]. An alternative explanation may involve transcriptomic dysregulation. Given that the *NR2E3* gene codes for a transcription factor, it might also modulate the expression of genes critical not only for rod photoreceptor differentiation and viability but also for retinal cell adhesion, synaptic structure organization, and ionic homeostasis [16,17]. For example, a reduction in *Crb1* expression—crucial for retinal lamination and cell adhesion—was noted in the naturally occurring *Nr2e3* mutant mouse model, as reported by Haider et al. [18]. Although numerous clinical features exist for distinguishing these conditions (such as the type of ERG abnormality, the presence of nyctalopia, pigmentary deposits, inheritance pattern, and others), accurately distinguishing between them remains difficult in certain individuals. Park et al. quantified retinal layer thickness as an approach to infer the location of macular schisis in XLRS and ESCS in order to improve differentiating between these conditions. In their cohort, macular schisis was predominantly located in the inner nuclear layer (INL) of XLRS subjects and in the outer plexiform layer and outer nuclear layer (OPL+ONL) of ESCS subjects. In cases of uncertain diagnosis, these structural differences may aid in differentiating between XLRS and ESCS [13]. The two genes together, *RS1* and *NR3E3*, were responsible for 33% (19/57) of all cases in our patient cohort.

A key distinction lies in differentiating CME, often associated with RP, from non-CME lesions or macular cysts seen in conditions such as XLRS and ESCS. On OCT, CME tends to show asymmetrical cystoid spaces, whereas macular cysts present a more symmetrical pattern. In CME, fluid typically accumulates in the inner nuclear layer, whereas macular cysts tend to localize to the outer plexiform layer. Moreover, the presence or absence of fluorescein leakage on FA can provide insights into the underlying mechanisms, with some CME cases in RP exhibiting minimal leakage, suggesting RPE or Müller cell dysfunction rather than BRB breakdown. It is noteworthy that cystoid spaces are located in macular regions where the outer retina remains relatively well preserved and the ellipsoid zone (EZ) can be identified [13,19,20].

An important distinguishing feature between CME with vascular etiology and macular cysts associated with IRDs is the relationship between visual acuity and OCT findings. In IRDs, BCVA does not correlate with neurosensory retinal thickness, in contrast to other CMEs, where such a correlation is often observed due to the underlying vascular etiology [21,22]. Moreover, the relationship between CME, BCVA, and foveal thickness in RP is not always predictable, as visual acuity often depends on the integrity of the EZ and external limiting membrane, critical structures for photoreceptor function, rather than solely on foveal thickness measurements [23]. However, some studies do show a correlation between CRT and BCVA values, but the prediction of visual function can further be strengthened with a more detailed evaluation of the outer retinal thickness, photoreceptor outer segment, EZ, and ELM measurements [23]. In our RP patients, only a weak correlation between BCVA and CRT could be detected (r_S_ = 0.36). Furthermore, BCVA in XLRS and ESCS is also more closely related to the integrity of the outer retinal structures (EZ, photoreceptor outer segment integrity) than to the size of the schisis cavity [24]. In our study, CRT and BCVA did not show a meaningful correlation in the non-CME CML and in the XLRS subgroup either, as expected. This highlights the importance of preserving outer retinal integrity in these conditions, as visual function can remain relatively stable even in the presence of severe schisis if the photoreceptors are intact. Therefore, a comprehensive OCT analysis that considers both structural and functional parameters is essential for assessing visual potential and guiding treatment decisions in RP patients with CML.

OCT angiography (OCTA) helps detect retinal neovascularization by assessing retinal microvasculature and is valuable for diagnosing CME in conditions such as age-related macular degeneration or diabetic macular edema [25]. Iovino et al. [26] found that although OCTA aids in evaluating retinal and choroidal blood flow in IRDs, its use is challenging due to artifacts. OCT and OCTA can identify alterations in retinal layers and capillaries, locating damage in XLRS cases. Studies also show that female carriers with normal acuity exhibit subtle OCTA-detected changes possibly due to lyonization [27]. The role of OCTA in managing IRDs is uncertain, and it is not routine in clinical exams for IRD patients.

According to the literature, CME is observed in 10–50% of RP patients [20,28,29]. The underlying mechanisms of CME linked to RP are not fully understood, with a genetic predisposition suspected as a contributing factor. Genetic mutations causing RP contri- bute to retinal degeneration, which then leads to secondary processes culminating in CME. Thus, CME in RP is mainly a secondary manifestation of the retinal degeneration rather than directly caused by specific gene mutations. Nevertheless, there is a paucity of information in the literature regarding the occurrence of CME across various genotypes and inheritance patterns in RP [30]. Some studies report that CME is generally unaffected by the inheritance patterns of RP. In a study conducted with 124 individuals, 38% presented with CME in at least one eye, with no discernible variation among autosomal recessive, autosomal dominant, isolated, or Usher II categories [8]. Nonetheless, certain genotypes are more frequently associated with the condition, such as *USH2A* mutations in autosomal recessive RP and *RHO* mutations in autosomal dominant RP. Conversely [31], some studies have not reported any instances of cystoid maculopathy in cases of X-linked recessive RP [8], highlighting its rarity within this subgroup. On the other hand, Pisani et al. [32] reported recently that CME was identified in 24.5% of their RP patients, of which 67% had CME in both eyes. Among the 60 different genes associated with RP found in their cohort, the most common genes were *RPGR*, *USH2A*, *RHO*, *RP1*, *RP2*, *PDE6B*, *PRPF31*, *NR2E3*, *RDH12*, *SNRNP200*, *PRPF8*, *PRPF3*, *CNGB1*, *EYS,* and *PRPH2*. An increased prevalence of CME, compared to the overall cohort, was noted for the genes *PRPF8* (72.7%), *PRPF3* (60%), *RHO* (56%), and *SNRNP200* (54.5%), all of which demonstrate an autosomal dominant inheritance pattern. Conversely, a decreased prevalence was observed in association with the genes *RDH12* (6.6%), *RPGR* (4.9%), and *RP2* (2.9%) relative to the total cohort. Another study by Gallo et al. demonstrated, that CME could be detected in 16.3% in a large cohort of patients with Usher syndrome [33].

In our RP patient cohort, the most common genotypes associated with CME included *USH2A*, *PRPF31*, *RPGR,* and *RHO*, which resembles the results of the abovementioned studies [33]. The incidence of CME was estimated at 12.4% in all RP cases and was more prevalent in AD-RP cases (34%), where CME manifested in 21% of all *RHO* cases, in 50% of *PRPF31* cases, and in 66% of *SNRNP200* cases. In contrast, in the AR-RP and XLRP cohorts, the occurrence of CME was less frequent, with incidences of 20% and 14%, respectively. Specifically, XLRP linked to *RPGR* mutations showed an incidence of 9%, which was somewhat higher than in other studies, but *USH2A*-related CME was comparable (16.6%) [32,33]. Conversely, no occurrences were documented in IRD cases attributed to *ABCA4*, *RPE65,* and *PRPH2*, despite these cases comprising a significant portion of the overall cohort.

The current study has some limitations, primarily its retrospective design, which resulted in the exclusion of certain patients due to missing genetic or imaging data. Furthermore, the utilization of standardized OCT imaging could have significantly improved the quality of the analysis, as it is acknowledged that all imaging techniques are subject to various artifacts [34]. The implementation of a prospective study design may facilitate a more detailed and targeted analysis of layer segmentation in OCT images, which was unattainable with our retrospective data. Additionally, while several disease-causing genes were identified, the relatively small number of cases per gene limits the generalizability of genotype-specific observations.

The treatment for CML in IRDs includes various pharmacological agents targeting fluid build-up and retinal dysfunction. Carbonic anhydrase inhibitors (CAIs), available orally and topically, are central to CME management by inhibiting fluid transport enzymes, reducing foveal thickness, and improving visual acuity [21]. Topical CAIs such as dorzolamide and brinzolamide are preferred in the first line due to fewer side effects [35]. Oral CAIs, such as acetazolamide, may be more effective but have systemic side effects such as fatigue and potential kidney stones [36]. CME recurrence with CAIs requires ongoing monitoring. Corticosteroids are also used to fight inflammation and edema, available in oral, parenteral, topical, and intravitreal forms. Intravitreal triamcinolone acetonide (IVTA) and dexamethasone implants (Ozurdex) reduce CRT and improve vision, but side effects such as cataracts limit their long-term use, and CME may recur [36]. Anti-VEGF agents emerged as another option, targeting VEGF to reduce vascular leakage and macular edema, although their efficacy in IRDs is variable and further research is needed [35]. Treatment aims to improve vision and preserve photoreceptor function and retinal integrity, reducing the risk of cell death and vision loss [37]. Managing CML is vital for optimizing outcomes of gene therapies by maintaining retinal stability [38].

## 5. Conclusions

CMLs pose a significant challenge in IRDs. Managing them requires advanced imaging, tailored treatments, and ongoing research to improve visual outcomes and retinal health. Current treatments provide relief but do not target the genetic causes of IRDs. Gene therapy shows promise for correcting genetic issues, but CML may interfere by disrupting retinal structure and gene delivery. Effective CML management is crucial for optimizing gene therapy and other treatments [37].

The present study evaluated the correlations between visual acuity and retinal structure across various IRD subtypes, highlighting that the integrity of the outer retina, as opposed to the overall thickness of the retina, is essential for the preservation of vision. The study also reviewed prevalence and genotype associations of CME in RP, with higher incidence in autosomal dominant forms.

## Figures and Tables

**Table 1:** Clinical characteristics of patients with CML and in the two subgroups, CME and non-CME CML. BCVA = best corrected visual acuity; CML = cystoid macular lesion; CRT = central retinal thickness, r_S_ = Spearman’s correlation coefficient; and SD = standard deviation.

Group	Patients (*n*)	%	Mean BCVA (Decimal ± SD)	Mean CRT (µm ± SD)	Correlation Between BCVA and CRT (r_S_)
CME	32	56%	0.43 ± 0.3	401 ± 182	0.36
Non-CME	25	44%	0.40 ± 0.2	465 ± 258	−0.05
Total cohort	57	100%	0.42 ± 0.26	430 ± 220	0.34

**Table 2:** Inheritance patterns and clinical characteristics in patients with cystoid macular lesions (CML).

Inheritance Pattern	Patients (*n*)	% of Total	Bilateral CML *n* (%)	Unilateral CML *n* (%)
Autosomal Dominant	14	24.6%	9 (64.3%)	5 (35.7%)
Autosomal Recessive	24	42.1%	13 (54.2%)	11 (45.8%)
X-linked	19	33.3%	14 (73.7%)	5 (26.3%)
Total cohort	57	100%	36 (63.1%)	21 (36.9%)

**Table 3:** Clinical and genetic characteristics of patients with CME in the IRD cohort. This table summarizes genetic and ophthalmological findings of patients with CME associated with inherited retinal diseases (IRD). BCVA values are reported in decimal notation. CRT measurements are provided in micrometers (µm) for the right eye (OD) and left eye (OS). Grey cells indicate that the respective eye was not affected by CML. ACMG = American College of Medical Genetics and Genomics; BCVA = best corrected visual acuity, CRT = central retinal thickness, LP = likely pathogenic; OD = right eye; OS = left eye; P = pathogenic; and VUS = variant of uncertain significance. Grey boxes indicate the unaffected eye of each patient.

Patient ID	Gene	Age (ys)	Gender	BCVA OD	BCVA OS	CRT OD (µm)	CRT OS (µm)	Genetic Variants	ACMG Classification
26	*DHDDS*	48	F	0.5	0.9	409	376	c.124A>G, p.(Lys42Glu) homozygous	P
27	*IFT140*	43	M		0.5		322	c.1565G>A, p.(Gly522Glu) c.3788C>T, p.(Pro1263Leu)	P VUS
28	*PDE6B*	32	M	0.15	0.016	265	262	c.385G>A, p.(Glu129Lys) homozygous	P
29	*USH2A*	32	F	1.0	1.0	349	365	c.8682-9A>G c.2299del, p.(Glu767Serfs*21)	P P
30	*USH2A*	37	F	0.3	0.2	421	547	c.11048-2A>G c.7595-2144A>G	P P
31	*USH2A*	27	M	0.6	0.6	754	762	c.7595-2144A>G c.582del, p.(Leu194Phefs*5)	P LP
32	*USH2A*	67	M		0.3		319	c.13335_13347delinsCTTG, c.1840G>T, p.(Gly614*)	P LP
33	*USH2A*	21	F	0.6	0.7	507	591	c.11864G>A, p.(Trp3955*) c.9424G>T, p.(Gly3142*)	P P
34	*USH2A*	30	F	0.8	0.6	366	345	c.11864G>A, p.(Trp3955*) c.(4627+1_4628-1)_(4987+1_4988-1)del	P P
35	*USH2A*	62	F		0.01		197	c.11864G>A, p.(Trp3955*) c.14621C>G, p.(Ser4874*)	P LP
36	*RPGR*	51	M		0.1		184	c.2293G>T, p.(Glu765*) hemizygous	P
37	*RPGR*	23	M		0.25		565	c.1415-9A>G hemizygous	P
38	*RPGR*	59	F	0.3	0.4	587	524	c.3067G>A, p.(Gly1023Arg) heterozygous	VUS
39	*RPGR*	46	M	0.25	0.3	277	296	c.2237_2238del, p.(Glu746Glyfs*23) hemizygous	P
40	*SNRNP200*	41	F	0.5	0.4	483	553	c.2599G>A, p.(Gly867Ser) heterozygous	VUS
41	*SNRNP200*	44	F	0.016		334		c.2599G>A, p.(Gly867Ser) heterozygous	VUS
42	*RHO*	19	M	0.6	0.6	834	838	c.1040C>T, p.(Pro347Leu) heterozygous heterozygous	P
43	*RHO*	58	M	1.0	0.3	349	308	c.541G>A, p.(Glu181Lys) heterozygous	P
44	*RHO*	31	F	0.2	0.2	525	935	c.50C>T, p.(Thr17Met) heterozygous	P
45	*RHO*	50	M	0.2	0.1	274	304	c.512C>T, p.(Pro171Leu) heterozygous	P
46	*PRPF31*	30	F	0.8	0.7	262	322	c.1040del, p.(Leu347Argfs*16) heterozygous	P
47	*PRPF31*	53	F	0.12		372		c.469C>T, p.(Gln157*) heterozygous	P
48	*PRPF31*	54	F	0.5	0.6	381	376	c.1040del, p.(Leu347Argfs*16) heterozygous	P
49	*PRPF31*	54	F	0.016		234		c.58G>T, p.(Gly20*) heterozygous	LP
50	*PRPF3*	21	F	0.7	1.0	396	336	c.1481C>T, p.(Thr494Met) heterozygous	P
51	*BEST1*	40	F	0.25		198		c.203A>G, p.(Tyr68Cys) heterozygous	LP
52	*BEST1*	31	F	0.5	0.5	208	217	c.920C>A, p.(Thr307Asn) heterozygous	LP
53	*CHM*	36	M		0.5		401	c.525_526del, p.(Glu177Lysfs*6) hemizygous	P
54	*CHM*	47	M	0.3	1	494	382	c.534del, p.(Glu179Lysfs*18) hemizygous	LP
55	*CHM*	38	M		0.5		292	c.1153del, p.(Gln385Serfs*24) hemizygous	P
56	*EFEMP1*	49	F		0.1		172	c.1033C>T, p.(Arg345Trp) heterozygous	P
57	*TTLL5*	50	M	0.2		119		c.2132_2135dup, p.(Met712Ilefs*15) c.1058A>G, p.(Asp353Gly)	P VUS

**Table 4:** Clinical and genetic characteristics of patients with non-CME (schisis-like) CML in the IRD cohort. This table summarizes the genetic and ophthalmological findings of patients with non-CME CML associated with inherited retinal diseases (IRD). BCVA values are reported in decimal notation. CRT measurements are provided in micrometers (µm) for the right eye (OD) and left eye (OS). Grey cells indicate that the respective eye was not affected by CML. ACMG = American College of Medical Genetics and Genomics; BCVA = best corrected visual acuity, CRT = central retinal thickness, LP = likely pathogenic; OD = right eye; OS = left eye; P = pathogenic; and VUS = variant of uncertain significance. Grey boxes indicate the unaffected eye of each patient.

Patient ID	Gene	Age (ys)	Gender	BCVA OD	BCVA OS	CRT OD (µm)	CRT OS (µm)	Genetic Variants	ACMG Classification
1	*MFRP*	20	M	0.3	0.25	476	485	c.498dup, p.(Asn167Glnfs*34) homozygous	P
2	*MFRP*	4	M		0.3		740	c.498dup, p.(Asn167Glnfs*34) homozygous	P
3	*C1QTNF5*	72	M		0.002		185	c.489C>A, p.(Ser163Arg) heterozygous	P
4	*RS1*	15	M	0.4	0.50	709	686	c.214G>A, p.(Glu72Lys) hemizygous	P
5	*RS1*	10	M	0.3	0.25	312	593	c.574C>T, p.(Pro192Ser) hemizygous	P
6	*RS1*	10	M	0.6	0.50	392	391	c.214G>A, p.(Glu72Lys) hemizygous	P
7	*RS1*	7	M	0.4	0.25	443	485	c.214G>A, p.(Glu72Lys) hemizygous	P
8	*RS1*	30	M	0.2	0.50	219	702	c.665dup, p.(Cys223Valfs*41) hemizygous	P
9	*RS1*	27	M	0.4	0.4	334	323	c.665dup, p.(Cys223Valfs*41) hemizygous	P
10	*RS1*	20	M	0.15	0.125	483	792	c.214G>A, p.(Glu72Lys) hemizygous	P
11	*RS1*	24	M	0.5	0.5	268	306	c.527T>C, p.(Phe176Ser) hemizygous	P
12	*RS1*	19	M	0.7	0.7	379	341	c.522+1G>A hemizygous	P
13	*RS1*	5	M	0.6		441		c.527T>C, p.(Phe176Ser) hemizygous	LP
14	*RS1*	19	M	0.8	0.4	561	618	c.574C>T, p.(Pro192Ser) hemizygous	P
15	*RS1*	76	M	0.25	0.32	449	528	c.610C>A, p.(Leu204Met) hemizygous	VUS
16	*NR2E3*	63	F	0.25	0.3	200	265	c.62T>G, p.(Leu21Arg) homozygous	P
17	*NR2E3*	69	F	0.25		257		c.119-2A>C homozygous	P
18	*NR2E3*	67	F	0.6	0.8	347	278	c.227G>A, p.(Arg76Gln) c.352G>C, p.(Val118Leu)	P LP
19	*NR2E3*	37	F	0.016	0.1	1614	1093	c.119-2A>C homozygous	P
20	*NR2E3*	32	M	0.3	1.0	336	328	c.481del, p.(Thr161Hisfs*18) c.932G>A, p.(Arg311Gln)	P P
21	*NR2E3*	30	F	0.6	0.9	446	417	c.227G>A, p.(Arg76Gln) homozygous	P
22	*NR2E3*	76	M		0.5		451	c.227G>A, p.(Arg76Gln) homozygous	P
23	*CRB1*	28	F		0.1		347	c.2843G>A, p.(Cys948Tyr) c.2101C>T, p.(Pro701Ser)	P VUS
24	*CRB1*	32	M	0.02		245		c.2843G>A, p.(Cys948Tyr) c.2101C>T, p.(Pro701Ser)	P VUS
25	*CRB1*	44	F	0.5		291		c.498_506del, p.(Ile167_Gly169del) c.2843G>A, p.(Cys948Tyr)	P P

## Data Availability

The data presented in this study are available on request from the corresponding author. The data are not publicly available due to privacy or ethical restrictions.

## Abbreviations

AD	autosomal dominant
AR	autosomal recessive
BCVA	best corrected visual acuity
BRB	blood retina barrier
CAI	carbonic anhydrase inhibitors
CME	cystoid macular edema
CML	cystoid macular lesion
CRT	central retinal thickness
ESCS	enhanced S-cone syndrome
EZ	ellipsoid zone
FA	fluorescein angiography
ILM	internal limiting membrane
INL	inner nuclear layer
IRD	inherited retinal disease
IVTA	intravitreal triamcinolone acetonide
LP	likely pathigenic
NGS	next generation sequencing
OCT	optical coherence tomography
OCTA	OCT angiography
OD	right eye
OS	left eye
ONL	outer nuclear layer
OPL	outer plexiform layer
P	pathogenic
RP	retinitis pigmentosa
RPE	retinal pigment epithelium
SD	standard deviation
VEGF	vascular endothelial growth factor
VUS	variant of uncertain significance
XLRP	X-linked retinitis pigmentosa
XLRS	X-linked retinoschisis
